# Genome Sequence of *Diabrotica virgifera virgifera virus* *2*, a Novel Small RNA Virus of the Western Corn Rootworm, *Diabrotica virgifera virgifera* LeConte

**DOI:** 10.1128/genomeA.00365-17

**Published:** 2017-05-18

**Authors:** Sijun Liu, Yuting Chen, Thomas W. Sappington, Bryony C. Bonning

**Affiliations:** a Department of Entomology, Iowa State University, Ames, Iowa, USA; b Corn Insects and Crop Genetics Research Unit, USDA, ARS, Ames, Iowa, USA

## Abstract

The genome of a novel small RNA virus, tentatively named *Diabrotica virgifera virgifera virus 2* (DvvV2), was identified in the western corn rootworm, *Diabrotica virgifera virgifera* LeConte, through transcriptome sequencing and confirmed by reverse transcription-PCR. Here, we report the near-complete nucleotide sequence and the genome organization of DvvV2.

## GENOME ANNOUNCEMENT

Western corn rootworm (WCR), *Diabrotica virgifera virgifera* LeConte (*Coleoptera*: *Chrysomelidae*), is a significant pest on maize in the United States, causing severe economic loss, and now threatens Europe (1). Given the resistance of this species to many current control methods (2), alternative approaches for management are required. To identify viruses that infect WCR, total RNA was extracted from adults and larvae collected from various locations in the United States and in Europe, and from virions isolated from field-caught insects as described previously (3). All libraries were sequenced using an Illumina HiSeq 2000 platform, generating 3.7 to 21 million single-end 100-bp reads. After trimming adaptor sequences, the Illumina reads were *de novo* assembled using Trinity (4). Contigs (≥200 nucleotides [nt]) were used for BLAST annotation against the NCBI nonredundant protein database. Here, we report the genome sequence of a novel unclassified small RNA virus, *Diabrotica virgifera virgifera virus 2* (DvvV2), the second small RNA virus identified from this analysis (3). The presence of the virus in WCR adults was confirmed by reverse transcription (RT)-PCR using SuperScript Ш (Life Technologies, Inc.) with overlapping primers, and rapid amplification of cDNA ends (SMARTer RACE cDNA amplification kit, Clontech).

The positive-sense, single-stranded RNA genome identified comprises 10,465 nt, excluding the poly(A) tail, and encodes a 3,427-amino acid polyprotein (nt 76 to 10386). The genome is AT-rich (57.7%). A 75-nt 5′ untranslated region (UTR) was confirmed by RT-PCR, while the 3′ UTR comprises 79 nt, followed by the poly(A) tail, as confirmed by 3′ RACE.

In contrast to other classified small RNA viruses of insects (5), the nonstructural proteins are encoded in the N-terminal region of the DvvV2 genome, and structural proteins are encoded at the C-terminal end. The polyprotein is most similar to that of *Solenopsis invicta virus 3* (SINV3, YP_002790880.2) (6) with 32% sequence identity across 68% of the nonstructural polyprotein sequence, and 31% identity across 71% of the structural polyprotein sequence. The two viruses also have a similar genomic structure. The DvvV2 polyprotein has similarity to *Moyer virus* (MoV, AOC55061.1), an unclassified virus, *Nylanderia fulva virus 1* (NFV1, YP_009268643.1), an unclassified ssRNA virus isolated from the tawny crazy ant (7), and *kelp fly virus* (KFV, YP_415507.1) (8). The protein sequence identity between DvvV2 and NFV1 is 26% with 84% sequence coverage. Conserved domains include nonstructural protein domains (RNA_helicase at amino acids 579 to 686) and RdRP_1 (amino acids 1874 to 2355). Two conserved protease motifs (GxCG and GxHxxG) were identified (amino acids 1568 to 1571 and 1588 to 1593) with the GxCG motif sequence (GDCGLPY), which is identical to those of SINV3, MoV, KFV, and NFV1. In contrast to iflaviruses and dicistroviruses, common small RNA virus structural domains, such as the Rh_v-like domain, were absent from DvvV2. Sequences of DvvV2 were detected by RT-PCR from WCR samples collected in Europe and in Arizona, Pennsylvania, Iowa, and Colorado, suggesting that this virus is widely distributed within WCR populations in the United States.

### Accession number(s).

The genome sequence was deposited in GenBank under accession number KY070327.
